# Draft Genome Sequences of Two *Pasteurella multocida* Strains Isolated from Buffaloes in India with Hemorrhagic Septicemia Disease

**DOI:** 10.1128/genomeA.00798-14

**Published:** 2014-08-07

**Authors:** J. E. Abrahante, B. M. Veeregowda, S. S. Hogtapur, R. E. Briggs, S. K. Maheswaran, S. Sreevatsan

**Affiliations:** a Department of Veterinary and Biomedical Sciences, University of Minnesota, St. Paul, Minnesota, USA; b Department of Microbiology, Veterinary College, Hebbal, Bangalore, India; c National Animal Disease Center, Agricultural Research Service, U.S. Department of Agriculture, Ames, Iowa, USA; d Department of Population Medicine, University of Minnesota, St. Paul, Minnesota, USA

## Abstract

*Pasteurella multocida* serotype B:2 is the causative agent of hemorrhagic septicemia in cattle and buffaloes in Asia. It is an acute fatal disease and is considered one of the most economically important diseases in this region of the world. We present here the draft genome sequences of strains 2213 and 3213 of *P. multocida*.

## GENOME ANNOUNCEMENT

Hemorrhagic septicemia (HS) is an acute, fatal, septicemic disease of cattle, buffaloes, and occasionally other species, caused by strains of two specific serotypes B:2 (Asian serotype) and E:2 (African serotype) of the bacterium *Pasteurella multocida*. However, HS-associated B:2 serotypes may occur in Africa, and E:2 serotypes may be found in Asia. Buffaloes are generally more susceptible than cattle. Asia and Africa are the regions with the highest endemic prevalence. Outbreaks of HS occur as catastrophic epizootics through high mortality and morbidity, and the disease is ranked as one of the most notable contagious diseases with the greatest economic importance (1, 2). In the United States, the disease was reported among bison in national parks in 1912, 1922, and 1965–1967. Outbreaks were also reported among dairy cattle and beef calves in 1969 and 1993 (1). However, apart from these sporadic outbreaks, the disease is not existent in the United States. A feature of this disease is the rapid spread of infecting bacteria from the respiratory tract to the blood and lymph to cause a fatal septicemia in less than 48 h. Death is caused by endotoxemia and intravenous inoculation of cattle and buffaloes with purified endotoxin from *P. multocida* isolated from HS-disease-reproduced clinical signs and death (3, 4).

The strains 2213 and 3213 of *P. multocida* were isolated from the blood of carcasses of two buffaloes (*Bubalis bubalis*) that died during outbreaks of HS disease from two different farms in South Karnataka, India. Both isolates belonged to capsular serogroup B as determined by using the multiplex capsular PCR system (5). Genomic DNAs of both strains were isolated in India using the Qiagen DNA extraction kits. Whole-genome sequencing was performed using the Illumina MiSeq platform. A total of 6,253,700 and 5,550,478 paired-end 150-bp reads of each genome were assembled into 34 and 32 contigs, respectively, for strains 2213 and 3213, using Velvet assembly software available on the Galaxy Suite (Minnesota Supercomputing Institute). Major gaps (>50 bp) were resolved on the GenBank assembly algorithm in an iterative manner in order to obtain a single assembled whole genome sequence. An annotation file was generated by Rapid Annotations using Subsystems Technology (http://rast.nmpdr.org/rast.cgi), and cross-verified using a reannotation performed by the Genbank annotation resource. RAST annotation of strain 2213 using strain 36950 as a reference produced a full genome of 2,309,333 bp carrying 2,163 coding sequences classified into 425 metabolic or virulence subsystems and 59 predicted RNAs. Strain 3213 produced a genome or 2,307,438 bp carrying 2,160 coding sequences that were classified into 424 subsystems and 57 predicted RNAs.

### Nucleotide sequence accession numbers.

The whole-genome sequence of *P. multocida* strains 2213 and 3213 have been deposited at the GenBank under accession numbers JNOK00000000 and JNOL00000000, respectively.
